# Pre- and Probiotics to Increase the Immune Power of Colostrum in Dogs

**DOI:** 10.3389/fvets.2020.570414

**Published:** 2020-11-06

**Authors:** Salvatore Alonge, Giulio Guido Aiudi, Giovanni Michele Lacalandra, Raffaella Leoci, Monica Melandri

**Affiliations:** ^1^Società Veterinaria “Il Melograno” srl, Varese, Italy; ^2^Department of Veterinary Medicine, University of Bari “Aldo Moro”, Bari, Italy

**Keywords:** colostrum, immunoglobulins, prebiotics, probiotics, pregnancy, bitch, dog, puppy

## Abstract

Wide differences in Ig concentration in canine colostrum have been reported. Thus, some litters can be at risk of passive immune transfer failure. Present study evaluated if supplementation with MOS, FOS, *E. faecium* and *L. acidophilus* along pregnancy increases colostrum quality. Twenty Great Dane bitches were divided into 4 groups. Control group (CG) received standard diet, only. Diet was supplemented with pre- and probiotics in other 3 study groups during: the last (1WG), last 2 (2WG), and last 4 (4WG) weeks of pregnancy, until parturition. Serum samples were collected at estrous (T0), supplementation beginning (T1), and parturition (T2). Colostrum was collected at C-section end. The IgG, IgM, and IgA were assayed on both matrices. In serum, IgG were higher at T0 than at parturition in all study groups and they significantly lowered from T0 to T1 in all groups. In colostrum, IgG and IgM were significantly higher in 4WG, while IgA already increased in 2WG group. Four-week pre- and probiotic supplementation resulted in the best immune properties of colostrum, as by the higher IgG, IgM, and IgA colostrum levels found in 4WG. Further studies would verify the exact mechanisms involved: pre-partum IgG mammary accumulation and B-cells GALT proliferation and mammary transfer. Further trials would verify whether these beneficial effects of pre- and probiotics on colostrum also lead to improved clinical conditions and immunological functions of newborns and puppies.

## Introduction

The immune system of puppies is functionally immature at birth. It already has in place all its constitutive functional components, which are however yet naive and will develop under the environmental influence (1), building up the immune memory and reaching the balance between Th1 and Th2 immune responses (2). Canine placenta is of endotheliochorial type, a relatively impenetrable barrier, thus colostrum consumption plays a pivotal role in the maturation and development of the neonatal immune system (3). Colostrum represents a crucial source of passive immunity thanks to immunological factors, such as IgG, IgA, and IgM (4). The IgG concentrate from blood to the mammary gland (5, 6), while IgM and IgA are concentrated in colostrum by an enteromammary link that allows the plasma cells transfer from gut-associated lymphoid tissue (GALT) to the mammary gland (3). In mice, it was demonstrated that plasma cells originating from precursors in Peyer's patches migrate via blood to the mammary gland. The GALT is the major site of B-cells proliferation; gut flora is essential for the development of GALT, thus for the humoral immune response (3).

Prebiotics and probiotics could interact with the immune composition of mammary secretions in different ways: interfering with GALT to modify the distribution and relative abundance of specific immune responses in dogs; inducing higher luminal secretions of IgA in the small intestine; enhancing the Ig concentration in mammary secretions, by means of the immune entero-mammary link in monogastric species (7–11). As a consequence, puppies could be transferred higher levels of immunoglobulins (Ig).

Previous authors reported possible variation in the concentration of specific antibodies within the colostral immunoglobulins (1, 12). Wide physiological differences in the Ig concentration in colostrum have been described, thus some puppies/litters can be at risk of death due to failure or lack of passive immune transfer (4). Passive immune transfer should be maximized to reach proper Ig concentrations in puppies (4). The present study aimed at evaluating whether the dietary supplementation with mannan-oligosaccharides (MOS), fructo-oligosaccharides (FOS), *Enterococcus faecium* and *Lactobacillus acidophilus* during pregnancy could increase the Ig concentrations in the serum and colostrum of bitches.

## Materials and Methods

### Ethics

The study was performed in accordance with the animal welfare committee ethical guidelines and all procedures were carried out according to the Italian legislation on animal care (DL 116, 27/01/1992) and the European Guidelines on Animal Welfare (Directive 2010/63/EU). The informed consent to the whole procedure was obtained from the owners of the dogs. The study was approved by the Ethical Committee of University of Bari “Aldo Moro” (Italy), under protocol CESA-DIMEV Bari n. 20/19.

### Animals

Twenty Great Dane bitches (3–6 years; 55–68kg) were recruited in FCI (Fédération Cynologique Internationale)-recognized kennel, following strict rules concerning animal health and welfare. Health-related aspects involved in the present study are reported in the following paragraphs.

All the animals were housed indoors in identical environmental conditions during the complete assay period since the dog breeder does not sell puppies until they reach an age of ≥75 days. Bitches were regularly vaccinated according to the WSAVA 2015 Vaccination Guidelines (13), i.e., every third year against distemper (CDV), infectious hepatitis (CAV), parvovirosis (CPV2) and para-influenza virus (PiV) (Nobivac CEPPi, MSD Animal Health srl, Milano, Italy), and annually for selected non-core diseases, i.e., leptospirosisi and kennel cough (Nobivac L4 and Nobivac KC, MSD Animal Health srl, Milano, Italy). During the month before the expected heat, animals were checked for protective antibody titres against CDV, CPV2, and CAV (14) and dewormed with fenbendazole (Panacur Forte, MSD Animal Health srl, Milano, Italy). All the bitches enrolled in the study reported satisfactory protective antibody titres against the cited diseases.

Each dog underwent a clinical examination to be proven healthy, including a thorough history evaluation, as well as a female breeding soundness exam (with clinical and ultrasonographic evaluation of the reproductive organs) before the beginning of pregnancy in order to avoid the effects of maternal illness on perinatal health (15, 16). To prevent any BIAS resulting from the possible impact of concurrent infections on antibody levels in body fluids, the health status of enrolled animals was checked throughout the study, with favorable results at all time points.

### Breeding Management

The breeding management from estrous to parturition was similar in all the litters. The day of ovulation was identified when the plasma progesterone concentration ranged between 4 and 10 ng/mL (17, 18), as evaluated using an enzyme-linked fluorescent assay (MiniVidas, BioMerieux, Marcy l'Etoile, France). Bitches were mated once 48 h later (19) with males of proven fertility (20).

The day of delivery was estimated from the blood progesterone concentration during estrous (63 ± 1 days after ovulation) and confirmed by fetal biometry (21, 22). Fetal health was assessed by fetal heart rate (23). For all the patients, C-section was planned in consideration of the health of mother and puppies, owing to a previous history or to the prediction of troubles at parturition (15).

Elective C-section was performed at term, matching information obtained at estrous (identification of the ovulation by serum plasma progesterone concentration), during pregnancy (fetal biometry), and at term (decline of serum plasma progesterone concentration below 2 ng/ml) (15).

At C-section, no pre-medication was given; an intravenous catheter was placed; 5-min-pre-oxygenation and fluid therapy with Ringer lactate 5 mL/kg/h iv (Ringer Lattato, Bbraun MilanoSpa, Milano, Italy) were administered. Anesthesia was induced with alfaxalone 3 mg/kg iv (Alfaxan, Dechra Veterinary Products Srl, Torino, Italy). The anesthetic agent was administered via the iv-catheter titrated to effect to reach oro-tracheal intubation. Anesthesia was then maintained with isoflurane 2% (Vetflurane, Virbac, Milano, Italy) in oxygen 90–95%, delivered via an oxygen concentrator (Nuvo Mark 8, GCE Mediline, Malmo, Sweden). An open circuit Mapleson type C was used. Opioids (methadone 0.2 mg/kg im, Semfortan, Dechra Veterinary Products Srl, Torino, Italy) and NSAIDs (meloxicam 0.2 mg/kg im, Inflacam, Virbac, Milan, Italy) were administered only after the extraction of the last puppy (24).

At whelping, neonates were clinically examined and their body-weight was measured before the first suckling.

### Feeding

All the bitches were fed the same dosed commercial diet (Adult Maintenance, Nutrix Più srl, Castelraimondo, Italy) according to metabolic requirements for gestation and received water *ad libitum*. According to the European Pet Food Industry Federation (FEDIAF) 2016 Guidelines, the total daily food intake was calculated, based on maintenance energy requirements (MER, kcal/die). The MER was calculated for each dog, considering the 4k coefficients, which are represented by breed, attitude, physiologic conditions, and health (25).

Twenty bitches were enrolled in the present study. Only one pregnancy for each bitch was considered. They were distributed into one of the 4 study groups according to a randomization list, taking into account that groups should not report any statistically significant difference concerning age and parity of enrolled subjects. The aim of this grouping technique was to avoid any possible BIAS deriving from the influence of age and parity on the immune quality of colostrum, that, despite the paucity of data regarding their effect on canine colostrum quality, is well described in other species (26).

Bitches were divided into 4 groups. Each group included 5 subjects. The control group (CG, parity 1-3, age 3-6 years) received a standardized commercial diet, only. The diet was supplemented with commercial tablets including a mix of pre- and probiotics (Florentero, Candioli SRL, Beinasco, Italy) from day 56 of gestation (last week of pregnancy; one-week group, named 1WG; parity 1-3, age 4-6 years), from day 49 of gestation (last two weeks of pregnancy; two-week group, named 2WG; parity 1-3, age 3-6 years) and from day 35 of gestation (last four weeks of pregnancy; four-week group, named 4WG; parity 1-3, age 3-5 years). In all study groups, the proper day to begin the supplementation was identified as follows: the number of days of gestation were counted after the day of ovulation, estimated by plasma progesterone concentration.

The commercial supplement (Florentero, Candioli SRL, Beinasco, Italy) includes: prebiotics: fructo-oligosaccharides 40% + mannan-oligosaccharides 4.05%; probiotics 8.86 x 10^9^ CFU/g of supplement: *E. faecium* DSM 10663/NCIMB 10415 4b1707 2.80 x 10^8^ CFU/g + *L. acidophilus* CECT 4529 4b1715 8.58 x 10^9^ CFU/g. This supplement is formulated in 1.2 gram tablets. Consequently, each tablet contains: FOS 480 mg, MOS 48.6 mg, *E. faecium* 3.36 x 10^8^ CFU, *L. acidophilus* 1.03 x 10^10^ CFU. Animals received one tablet every 10 kg of bodyweight, following the manufacturer's instruction. The total daily dose calculated for each dog was divided in two administrations (8 a.m. and 8 p.m.), given directly into the oral cavity.

### Samples Collection and Analysis

Serum samples were collected at estrous monitoring (T0, basal serum), supplementation beginning (T1), and parturition (T2). Blood was collected from the cephalic vein in an empty plastic vial, samples were immediately centrifuged; then, serum samples were separated and moved to empty Eppendorf vials and frozen at −20°C until essay.

Colostrum was collected immediately at the end of the C-section, before the first suckling, after cleaning the mammary gland with an antimicrobial solution containing soap and chlorhexidine. Samples were obtained from the 4th and 5th teat of each line, by gentle manual milking, into four different empty Eppendorf vials. Literature reports a wide variability in the Ig content of colostrum milked from different teats but no factors affecting this phenomenon have been identified yet (27). Thus, colostrum was sampled from 4 different mammary glands of each bitch, Ig concentrations were assessed separately, and the mean concentration of each Ig class was calculated for every bitch.

All the samples were kept frozen at −20°C until the day of analysis and thawed at room temperature before the analysis. After thawing and before the analysis, colostrum was centrifuged (2000 x g for 30 min at 4°C) (27), to isolate the casein-free fat-free whey for further processing.

The IgG, IgM and IgA were assayed in serum and colostrum samples by commercial kits (Dog IgG-, IgM-, IgA ELISA, Immunology Consultants Laboratory Inc., Portland, USA) based on the principle of the double antibody sandwich ELISA. According to the expected results (28) and following the manufacturer's guidelines, samples were diluted with ELISA-sample-diluent. All the samples were assayed in duplicate and the mean value of the two assays was considered. The Ig (IgG, IgM, IgA) contained in the samples reacted with the respective anti-Ig antibodies, that were adsorbed to the surface of polystyrene microtitre wells. Unbound proteins were removed by washing. Then, anti-Ig antibodies conjugated with horseradish peroxidase were added. Such enzyme-labeled antibodies created complexes with the previously bound Ig (IgG, IgM, IgA). After a further washing passage, the enzyme bound to the immunosorbent was assayed by adding a chromogenic substrate, 3,3',5,5'-tetramethylbenzidine (TMB). The amount of the bound enzyme varied directly with the concentration of the Ig (IgG, IgM, IgA) tested. The absorbance, read at 450 nm by the microplate reader (Mindray MR96A, Shenzhen Mindray Bio-Medical Electronics co Ltd., Nanshan, China), was a measure of the Ig concentration in the tested sample. The amount of Ig (IgG, IgM, IgA) in the sample was finally interpolated from the standard curve obtained from the standards, and corrected for sample dilution.

### Statistical Analysis

All the IgG, IgM, and IgA concentrations obtained were reported on an Excel 2010 Office file, mean values ± SD were calculated for each parameter. The normality of data distribution was checked by the Shapiro-Wilk test, thus the statistical analysis was performed by ANOVA, and ANOVA Repeated Measures. In case of significant differences among sample groups, values were compared by the Tukey HSD test.

The litter size, the total number of puppies and litters in each study group, the male/female ratio, the maternal age and parity were statistically compared by ANOVA to check the absence of zootechnical differences among groups.

The concentration of IgG, IgM, and IgA in serum at T0, T1, and T2 were compared among the 4 study groups by ANOVA and within the 4 study groups by ANOVA Repeated Measures.

Finally, the concentration of IgG, IgM and IgA in colostrum was statistically compared among the 4 study groups by ANOVA.

For every Ig category, the coefficient of variation was calculated to express the variability of the corresponding Ig concentrations among different mammary glands within one bitch. The coefficient of variation was obtained for every bitch and expressed as percentage. Then, the mean value ± SD of the coefficient of variation was calculated.

For each study group and every Ig class, the percentage on total Ig in colostrum and the ratio between concentration in colostrum and in basal serum were calculated, expressed as mean value ± SD, and statistically analyzed among the 4 study groups by ANOVA.

Results were considered significant for *P* < 0.05. The statistical analysis was performed with the online tools VassarStats: Website for Statistical Computation (http://vassarstats.net, Vassar College, New York, NY, USA) and Social Science Statistics (https://www.socscistatistics.com, Jeremy Stangroom, USA).

## Results

All the bitches delivered full term alive puppies. All puppies were healthy (29) and birth-weight was within normal breed ranges (30).

No statistically significant differences among the 4 study groups were found concerning zootechnical parameters, such as the number of litters and puppies in each study group (Table 1), male/female ratio, litter size, maternal age, and parity.

**Table 1:** Number of puppies in each litter in the 4 study groups.

**Litters**	**1**	**2**	**3**	**4**	**5**	**N° total**	**Mean ± SD**
Control group	6	7	12	4	2	31	6.2 ± 3.77
1WG	1	10	13	2	4	30	6.0 ± 5.24
2WG	9	8	1	8	3	29	5.8 ± 3.56
4WG	7	5	9	4	7	32	6.4 ± 1.95

*No statistically significant differences were found within the mean number of puppies in the 4 study groups*.

In the maternal serum, IgG significantly decreased from T0 to T2 in all the study groups (CG: 538.53 ± 169.54 mg/dl vs. 302.19 ± 128.26; 1WG: 558.78 ± 138.59 vs. 320.40 ± 193.75; 2WG: 519.03 ± 61.06 vs. 318.05 ± 127.44; 4WG: 588.25 ± 55.82 vs. 366.50 ± 184.31), while IgA and IgM did not vary (Table 2).

**Table 2:** Ig concentration in serum of bitches enrolled in the 4 study groups at T0, T1, and T2.

**mg/dl**			
**Time**	**T0**	**T1**	**T2**
**IgG**			
Control group	538.53 ± 169.54^(a)^		302.19 ± 128.26^(b)^
1WG	558.78 ± 138.59^(a)^	414.45 ± 266.65^(ab)^	320.40 ± 193.75^(b)^
2WG	519.03 ± 61.06^(a)^	411.93 ± 221.52^(ab)^	318.05 ± 127.44^(b)^
4WG	588.25 ± 55.82^(a)^	443.10 ± 277.14^(ab)^	366.50 ± 184.31^(b)^
**IgM**			
Control group	131.78 ± 24.67^(a)^		130.71 ± 21.92^(a)^
1WG	132.05 ± 18.75^(a)^	127.08 ± 20.68^(a)^	154.37 ± 12.23^(a)^
2WG	128.75 ± 20.15^(a)^	135.87 ± 8.61^(a)^	148.35 ± 23.89^(a)^
4WG	133.70 ± 22.63^(a)^	127.33 ± 28.86^(a)^	143.46 ± 16.43^(a)^
**IgA**			
Control group	32.15 ± 7.31^(a)^		33.35 ± 2.98^(a)^
1WG	30.75 ± 1.45^(a)^	31.33 ± 0.58^(a)^	30.88 ± 1.87^(a)^
2WG	31.40 ± 2.65^(a)^	32.51 ± 3.94^(a)^	31.12 ± 3.23^(a)^
4WG	30.45 ± 4.05^(a)^	33.84 ± 2.95^(a)^	27.06 ± 9.03^(a)^

*T0, mating time; T1, diet supplementation beginning; T2, parturition. Different superscript letters denote statistical differences within rows for each Ig category. No statistically significant differences were found within columns for any Ig category*.

No statistical differences were observed concerning Ig concentrations (IgG, IgM as well as IgA) in maternal serum among the 4 study groups, either at the beginning of the study (T0), or at parturition (T2), even if a trend toward higher IgG concentrations at T2 was found with increasing supplementation length (Table 2). No significant differences were found for Ig serum concentrations among 1WG, 2WG, and 4WG at supplementation beginning (T1) (Table 2). Referring to the time point T1, the CG was not included in the comparison as bitches from CG, not receiving any supplementation, were never sampled between T0 and T2.

**Figure 1:**
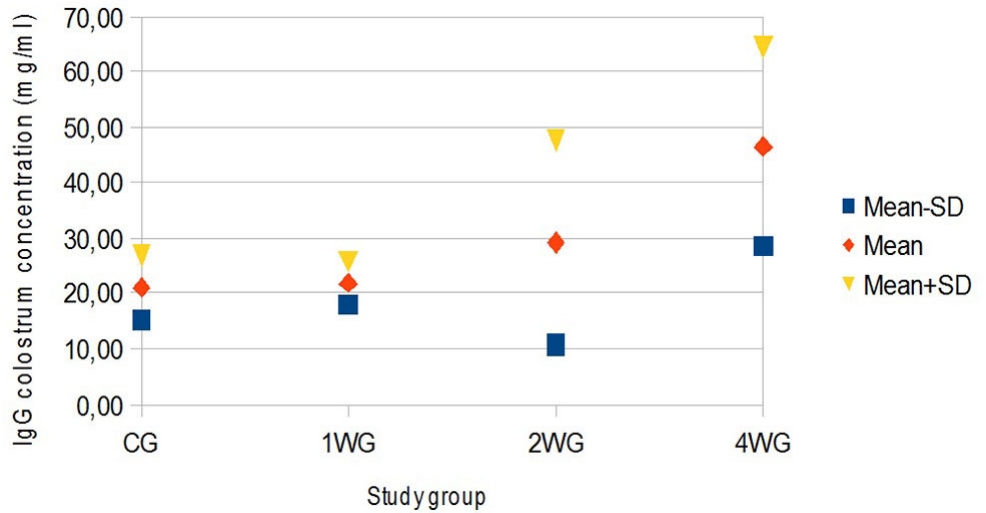
Variability of colostrum IgG concentration depending on study group. Results are presented as mean ± SD. CG, control group; 1WG, one-week group; 2WG, two-week group; 4WG, four-week group. Each study group enrolled 5 bitches.

**Figure 2:**
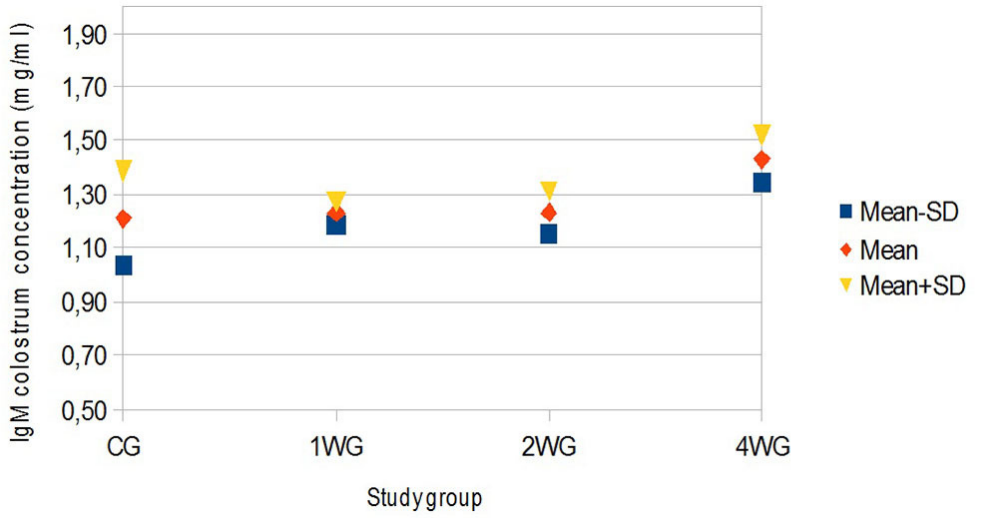
Variability of colostrum IgM concentration depending on study group. Results are presented as mean ± SD. CG, control group; 1WG, one-week group; 2WG, two-week group; 4WG, four-week group. Each study group enrolled 5 bitches.

**Figure 3:**
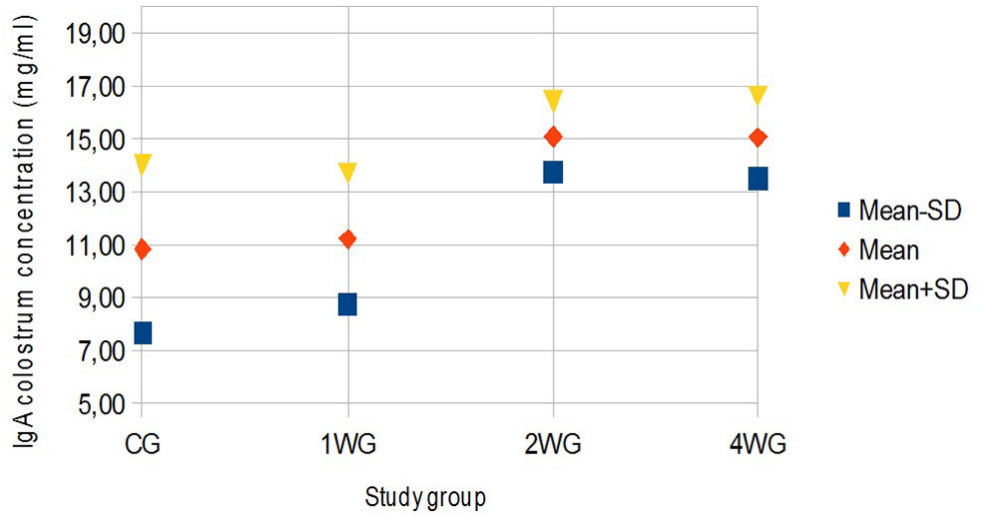
Variability of colostrum IgA concentration depending on study group. Results are presented as mean ± SD. CG, control group; 1WG, one-week group; 2WG, two-week group; 4WG, four-week group. Each study group enrolled 5 bitches.

In colostrum, IgA were significantly higher in 4WG (15.07 ± 1.57 mg/ml) and 2WG (15.10 ± 1.33 mg/ml) than in CG (10.84 ± 3.17 mg/ml) and 1WG (11.24 ± 2.47 mg/ml), while IgG and IgM were significantly more concentrated in 4WG, only (46.51 ± 17.85 mg/ml vs. CG 21.06 ± 5.83 mg/ml, 1WG 21.90 ± 3.88 mg/ml, 2WG 29.20 ± 18.36 mg/ml for IgG; 1.43 ± 0.09 mg/ml vs. CG 1.21 ± 0.18 mg/ml, 1WG 1.23 ± 0.04 mg/ml, 2WG 1.23 ± 0.08 mg/ml for IgM) (Table 3). A graphical representation of results obtained on colostrum is given in Figure 1 for IgG, Figure 2 for IgM, and Figure 3 for IgA.

**Table 3:** Ig concentration in colostrum of bitches enrolled in the 4 study groups.

**mg/ml (g/l)**	**IgG**	**IgM**	**IgA**
Control group	21.06 ± 5.83^(a)^	1.21 ± 0.18^(a)^	10.84 ± 3.17^(a)^
1WG	21.90 ± 3.88^(a)^	1.23 ± 0.04^(ab)^	11.24 ± 2.47^(ab)^
2WG	29.20 ± 18.36^(ab)^	1.23 ± 0.08^(ab)^	15.1 ± 1.33^(b)^
4WG	46.51 ± 17.85^(b)^	1.43 ±0.09^(b)^	15.07 ± 1.57^(b)^

*Different superscripts denote statistical differences within columns*.

The mean coefficient of variation of immunoglobulins concentrations in colostrum (sampled from four different teats of each bitch) was calculated as follows: 44.83 ± 27.48% for IgG, 10.81 ± 7.36% for IgM, and 19.64 ± 15.44% for IgA.

The percentage of each Ig category on total Ig in colostrum for every study group is reported in Table 4. No statistically significant differences among the 4 study groups were found for this index.

**Table 4:** Percentage of each Ig category on total Ig in colostrum for every study group.

**%**	**IgG/Total Ig**	**IgM/Total Ig**	**IgA/Total Ig**
Control group	63.69 ± 8.38 Heddle and Rowley (31): 79% Chastant and Mila (4): 60-75%	3.68 ± 0.86 Heddle and Rowley (31): 2%	32.62 ± 8.30 Heddle and Rowley (31): 20% Chastant and Mila (4): 16-40%
1 week	64.52 ± 7.73	3.77 ± 0.24	31.71 ± 7.49
2 weeks	60.08 ± 12.32	3.09 ± 1.18	36.83 ± 11.14
4 weeks	71.84 ± 9.32	2.45 ± 0.84	25.71 ± 8.52

*Referring to the control group, a comparison to the already existing literature is reported*.

*No statistically significant differences were found within columns*.

The ratio between concentration in colostrum and in basal serum for each Ig class for all study groups is described in Table 5. Referring to IgG and IgM, the ratio between concentrations in colostrum and in basal serum was statistically significantly higher in 4WG (7.00 ± 1.83 and 1.16 ± 0.20, respectively), only. Even if not statistically significant, a trend toward an increased ratio was already evident for IgG in 2WG (5.63 ± 3.43). Conversely, referring to IgA, the ratio between the concentrations in colostrum and in basal serum already significantly increased in 2WG (46.88 ± 4.22 in 2WG and 44.15 ± 4.21 in 4WG), with a visible trend in 1WG (34.06 ± 5.74).

**Table 5:** Ratio between concentration in colostrum and in basal serum for each Ig class for all study groups.

**Colostrum/basal serum**	**IgG**	**IgM**	**IgA**
Control group	3.88 ± 1.48^(a)^ Vaerman and Heremans, (32): 1.5 Heddle and Rowley, (31): 1.6 Ricks et al., (12): 3 Day et al., (1): 1.6 Chastant and Mila, (4): 2-3	0.87 ± 0.19^(a)^ Vaerman and Heremans, (32): 0.46 Heddle and Rowley, (31): 0.14 Ricks et al., (12): 0.57 Day et al., (1): 0.14 Chastant and Mila, (4): 0.15-0.25	31.53 ± 11.39^(a)^ Vaerman and Heremans, (32): 14 Heddle and Rowley (31): 5 Ricks et al., (12): 22 Day et al., (1): 5 Chastant and Mila, (4): 5-10
1 week	3.74 ± 0.10 ^(a)^	1.02 ± 0.11^(ab)^	34.06 ± 5.74^(ab)^
2 weeks	5.63 ± 3.43^(ab)^	0.93 ± 0.05^(a)^	46.88 ± 4.22^(b)^
4 weeks	7.00 ± 1.83^(b)^	1.16 ± 0.20^(b)^	44.15 ± 4.21^(b)^

*Referring to the control group, a comparison to the already existing literature is reported. Note that Day et al., (1) and Chastant and Mila, (4) are reviews, while Vaerman and Heremans (32) analysed data from 1 subject, Heddle and Rowley, (31) based their data on 2–4 dogs and Ricks et al., (12) referred to 6 bitches*.

*Different superscripts denote statistically significant differences within columns*.

## Discussion

During the whole study, all the bitches showed IgG, IgM, and IgA concentrations in serum within the normal range reported for the canine species (31).

The absence of any statistically significant difference among groups concerning zootechnical parameters was very relevant, allowing to avoid possible systematic BIAS due to parity and/or maternal age (26). Zootechnical parameters, i.e., maternal age and parity, were not addressed in the present study. However, the absence of any significant difference about them among groups needed to be statistically proven to avoid any possible BIAS, because in other species the effect of maternal age and parity on colostrum composition is well described, such as in swine (26, 33).

In the present study, the total immunoglobulin concentration in the colostrum in the control group (CG) was 33.11 mg/ml, thus above the minimum threshold reported for the first day of lactation (15 mg/ml) (31). In the present study, colostrum was sampled immediately at the end of the C-section, before the first suckling to avoid any possible BIAS. It has previously been described that at the beginning of lactation, the milk yield is low, but it increases greatly few hours after parturition. Hence, colostrum, initially rich in immunoglobulins, is gradually diluted in increasing mammary secretions (34, 35). Progressively, milk production increases, while IgG accumulated in the mammary gland do not, thus they are diluted in a higher volume and the IgG concentration lowers (27). Moreover, neither the breed nor the duration of parturition could affect this result as all the bitches were Great Danes and all the litters were born by timed C-section, always planned and performed with the same operating procedure.

The colostrum of all the bitches met satisfying immune quality: the IgG concentration in colostrum was always higher than 3.4 g/L (4). On the other hand, the immunological quality of colostrum was strongly influenced from the supplementation. In line with literature reports (4, 12, 27), the IgG concentration in the colostrum of the CG was approximately 4 times higher than in the basal serum; 1-week supplementation did not succeed in modifying this ratio, while in 2WG and in 4WG, it increased by more than 5.5 and 7 times, respectively (see Table 2 for basal serum IgG concentration, Table 3 for colostrum IgG concentration, and Table 5 for the ratio between colostrum and basal serum IgG concentration).

The basal concentration of IgG in the maternal blood was higher than at parturition in all the groups, accordingly to what happens in sows and cows, where IgG concentrate from blood to the mammary gland (5, 6). This process would mainly occur at the very end of gestation as no statistical differences were observed in the IgG serum level either between T0 and T1, or at the beginning of the supplementation (T1) among the different study groups. In humans, it has previously been reported that serum IgG are concentrated into the mammary gland by their link to specific receptors FcRn (Fragment constant Receptor neonatal) set on the apical aspect of the mammary cells (36) toward the end of pregnancy. Such a biomolecular process has never been demonstrated in dogs, but the existence of an analogous system could be hypothesized. Even if a statistically significant difference was not observed, it is interesting to note that the IgG concentration in serum at T2 seems to have a higher trend in subjects with a longer supplementation period. The likely mechanism can be found in the synergistic effect between pre- and probiotics and the functioning of the immune system. The longer the supplementation lasts, the more evident its effect becomes. Thus, higher IgG levels are produced by the maternal immune system, but, due to the pregnant status, the mammary gland works at its best as a sponge and accumulates the IgG available in blood as much as possible. The present results confirm that the concentration of IgG in colostrum was statistically influenced by the length of the supplementation period. An initial effect was already evident in the 2WG group, but it became statistically significant in the 4WG, only, reaching a mean concentration of 46.51 mg/ml, i.e., more than twice that of the control group. It could be hypothesized that only if IgG levels in blood would exceed the filtrating and accumulating capability of the mammary gland, a statistically significant higher concentration would remain in the blood and could be detected in serum samples.

Beside IgG, colostrum levels of IgM and IgA, too, were considered immune parameters for neonates protecting them either systemically, passing through the gut epithelium, or after gut closure, coating the epithelial layer (37). Protection depends on IgM and IgA specificity: produced by the mother, they are likely specific against environmental antigens (3, 38). According to literature (12), these results confirmed that IgA always had the highest colostrum to serum ratio in all study groups, but significantly higher by 2-week supplementation (Table 5). The IgM were generally found in higher concentrations in basal maternal serum than in the colostrum, whereas only the 4-week supplementation managed to significantly increase, thus reverse, this proportion (Table 5).

The results of this study detected an increase in IgM concentrations in the mammary secretions of the 4WG supplemented group without any concomitant significant increase in the maternal blood. The stimulation of the intestinal immune system by scFOS had already been described in dogs (3, 7). In particular, a higher rate of IgM synthesis in the immunological sites in the mammary gland itself was detected in bitches receiving a diet supplemented with fructo-oligosaccharides from the 35th day of gestation, in absence of IgM blood filtration through the mammary gland epithelium (3).

On the other hand, the 2-week-supplementation period succeeded in improving of colostrum IgA level. The effects of yeast-derived mannan-oligosaccharides (MOS) and fructo-oligosaccharides (FOS) have been studied in dogs (10, 39, 40). Those studies suggested that MOS and FOS could increase fecal *Lactobacillus* and *Bifidobacterium* and elevate ideal IgA concentrations (10, 41). Moreover, it was suggested that probiotics (especially lactic acid bacteria) can enhance adaptive and innate immune responses, including the local production of IgA (42, 43). In addition, the role of *E. fecium* was reported in a preferential IgA switch of mucosally primed B cells associated with an increase in the mucosal IgA response due to the specific homing of the IgA-producing B cells in the gut (44–46). Finally, it has previously been stated that the combined use of prebiotics and probiotics could have a cumulative effect on the regulation of gut immunity, including an increase in IgA production (47). Thus, as suggested by different authors, the immature B-cells initiated in the gut, not only accumulate within the gut lamina propria, but also in other organs such as the mammary gland, where they mature (48), supporting the existence of an immune entero-mammary link.

The administered supplementation, independently of its duration, never affected the relative abundance of different Ig categories in the colostrum of bitches; no statistically significant differences were found in the comparison of the percentages of single Ig classes on total Ig in colostrum among groups (Table 4).

Four-week pre- and probiotic supplementation resulted in the best immune properties of colostrum, as by the higher IgG, IgM, and IgA colostrum levels found in 4WG. Further studies are warranted to verify the exact mechanisms that could be involved: pre-partum IgG mammary accumulation and B-cells GALT proliferation and mammary transfer.

A recent clinical retrospective study suggested that pre- and probiotics administered to the mother during pregnancy improve clinical gastro-intestinal conditions in puppies (49). Further studies would be advisable to verify whether these beneficial effects of pre- and probiotics on colostrum also lead to improved clinical conditions and immunological functions of newborns and puppies.

## Data Availability Statement

The data that support the findings of this study are available from the corresponding author, upon reasonable request.

## Ethics Statement

The animal study was reviewed and approved by CESA-DIMEV Bari n. 20/19. Written informed consent was obtained from the owners for the participation of their animals in this study.

## Author Contributions

SA and MM: conceptualization, investigation, and writing–original draft. GA and RL: data curation. SA, RL, and MM: formal analysis. GA and GL: funding acquisition. SA: project administration and visualization. SA, GA, GL, and MM: resources. SA and GL: supervision. GA: validation. SA, GL, and MM: writing–review and editing. All authors contributed to the article and approved the submitted version.

## Conflict of Interest

MM and SA are employed by the company Società Veterinaria “Il Melograno” srl. The remaining authors declare that the research was conducted in the absence of any commercial or financial relationships that could be construed as a potential conflict of interest.
